# Quantifying Uncertainty in Laser-Induced Damage Threshold for Cylindrical Gratings

**DOI:** 10.3390/mi16010045

**Published:** 2024-12-30

**Authors:** Yuan Li, Junqi Xu, Guoliang Yang, Lihong Yang, Junhong Su

**Affiliations:** 1 School of Physics and Telecommunication Engineering, Shaanxi University of Technology, Hanzhong 723001, China; 2 School of Opto-Electronical Engineering, Xi’an Technological University, Xi’an 710021, China; jqxu2210@163.com (J.X.); yangguoliang@xatu.edu.cn (G.Y.); yanglihong@xatu.edu.cn (L.Y.)

**Keywords:** laser-induced damage threshold (LIDT), gratings, uncertainty of the LIDT, laser damage probability, laser fluence

## Abstract

The laser-induced damage threshold (LIDT) is a key measure of an optical component’s resistance to laser damage, making its accurate determination crucial. Following the ISO 21254 standards, we studied the measurement strategy and uncertainty fitting method for laser damage, establishing a calculation model for uncertainty. Research indicates that precise LIDT measurement can be achieved by using a small energy level difference and conducting multiple measurements. The LIDT values for the cylindrical grating are 15.34 ± 0.00052 J/cm^2^ (95% confidence) and 15.34 ± 0.00078 J/cm^2^ (99% confidence), demonstrating low uncertainty and reliable results. This strategy effectively measures the LIDT and uncertainty of various grating surface shapes, offering reliable data for assessing their anti-laser-damage performance.

## 1. Introduction

Quantifying uncertainty in laser-induced damage thresholds (LIDT) is crucial for the reliability of laser systems [1,2], particularly in the application of high-power laser systems [3,4,5]. With the rapid development of laser technology, optical elements are playing an increasingly important role in various high-power laser systems [6,7,8]. The laser damage resistance of optical elements is a key factor limiting the development of high-power laser systems [9,10]. The laser damage threshold (LIDT) of optical elements is an important index to evaluate the anti-laser-damage performance of optical elements [11,12,13,14]. The LIDT of optical elements depends on laser parameters and the operational mode [15,16,17,18,19]. Assessing the laser damage threshold of optical components is crucial for understanding their resistance to laser damage.

In recent years, the testing of the LIDT of optical components has been extensively researched [20,21,22]. Jensen et al. explore the statistical aspects of laser-induced damage threshold (LIDT) testing by employing continuously distributed energy values for sample irradiation and enhancing data processing methods to minimize uncertainties and errors in evaluations [20]. MStehlik et al. demonstrate that beam size affects the measurement of sub-picosecond intrinsic LIDT of dielectric oxide coatings, leading to relative uncertainty [23]. L. Lamaignere et al. examined the laser-induced damage thresholds of two coatings and demonstrated that systematic errors, measurement errors, and sample uniformity can influence the uncertainty in LIDT [24]. M. Chorel et al. quantified silicon oxide and haf-nium oxide single-layer films from various suppliers, emphasizing that precise meas-urement of beam energy density is crucial for determining LIDT [25]. 

However, conventional measurement methods have limitations in accuracy and repeatability [26,27], thus making it difficult to evaluate the LIDT of optical components with unique structures. Research on determining the LIDT for optical elements with structured flat surfaces is limited. Current studies primarily focus on testing methods for traditional optical components, with little attention given to the uncertainty of structured optical components. This paper aims to establish a process for determining the LIDT and fitting uncertainty for optical elements with periodic surfaces, thereby providing a reliable framework for accurately evaluating their LIDT.

This study combines experimental analysis with theoretical analysis. According to the standard ISO 21254 [28], a YAG laser with a wavelength of 1064 nm, a pulse width of 10 ns, a spot radius of 400 μm, and a maximum energy of 200 mJ irradiates the sample surface using a one-on-one method. First, experimental data on the LIDT of an optical element with cylindrical periodic structured surfaces are collected. Then, uncertainty is analyzed using statistical methods, and an evaluation of the LIDT uncertainty method for optical elements with periodic structured surfaces is proposed. This study will provide a new theoretical basis and practical guidance for the LIDT evaluation of optical components with periodic structured surfaces, and the results of this study will also provide a valuable reference for improving LIDT test methods and designing new optical components in laser technology.

## 2. Materials and Methods

### 2.1. Materials

**Figure 1:**
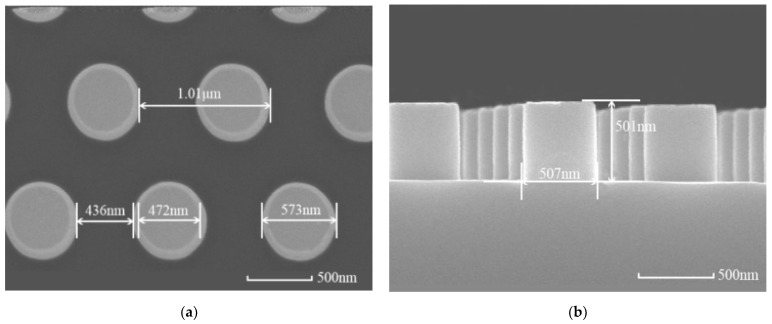
SEM images of the optical element with periodic cylindrical structured surfaces. (**a**) Top view; (**b**) profile view.

The sample is a two-dimensional grating surface with a periodic arrangement of cylindrical structures, fabricated by nanoimprinting and inductively coupled ion beam etching on a 500 nm thick silicon oxide film deposited via plasma-enhanced chemical vapor deposition on a 4-inch quartz glass substrate. Figure 1 shows Scanning Electron Microscope (SEM) images of the optical element with periodic cylindrical structured surfaces. The mean dimensions of the structured surface are as follows: the diameter d = 522.5 nm, the height h = 500 nm, and the period Λ = 1.01 μm. The processing error is within 10 nm. The error of the structure surface parameters is a microscopic quantity, and its uncertainty has no significant effect on the uncertainty of the LIDT value. The LIDT value is mainly affected by the factors related to the test process. Before the test, the sample was ultrasonically cleaned to ensure a clean surface.

### 2.2. Experimental Setup and Principles of the LIDT Test

**Figure 2:**
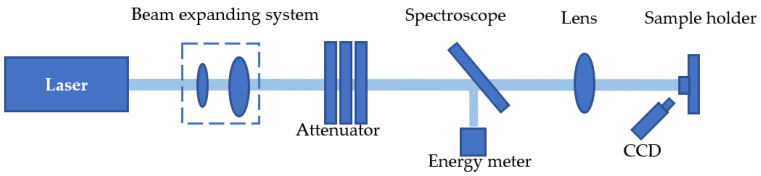
Schematic diagram of the laser-induced damage test system.

The laser-induced damage threshold of optical components with structured surfaces was determined using the 1-on-1 method, following the international standard ISO 21254. A 1064 nm pulsed laser with a 10 ns pulse width was employed for the measurements. Figure 2 is a schematic diagram of the laser-induced damage threshold (LIDT) test system [29,30,31,32].

**Figure 3:**
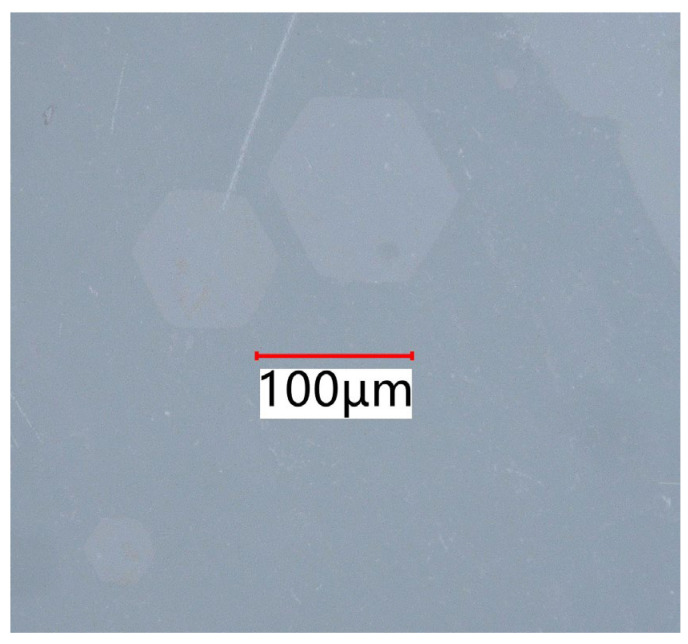
Typical morphology of damaged spots.

According to the standard ISO 21254-2 [33], the laser-induced damage threshold of the optical component is determined by the energy density at which damage has zero probability, denoted as the laser energy per unit area (J/cm^2^). The image recognition method was adopted to identify the damaged spots. The changes in the image before and after laser irradiation were compared to determine if the irradiation point was damaged. The laser damage threshold was tested according to the international standard ISO 21254-2, and a 1-on-1 zero-probability damage test method was adopted; that is, the laser irradiated the sample surface a single time, and a position could only be irradiated once. A location can only be irradiated once, and the spacing between adjacent locations ensures that each irradiation remains independent and does not interfere with others. The laser parameters include a 1064 nm pulse laser with a pulse width of 10 ns and an effective spot radius of 400 μm. Figure 3 illustrates the typical morphology of damaged points observed under a polarized light microscope (KEYENCE VHX-7000 series, Keyence Corporation, Osaka, Japan). The size of the damaged spots varies with energy. In the observable area, three distinct damage points are visible, with clear boundaries and a regular hexagonal shape.

The damage probability p = k/m, where k is the number of spots damaged at each energy level, and m is the total number of laser irradiations at this level. In principle, for planar thin-film elements, each energy level is usually tested 10 times. The anisotropy of the structure’s surface leads to significant errors in damage probability calculations when only a limited number of test points (10) are used at a given energy level. Therefore, it is crucial to maximize the number of tests at each energy level. This may explain the frequent failure of the flat film method previously used to measure the structure’s surface. The selected energy level ranges from a 0 to 100% probability of damage. This involves predicting the laser damage energy of the sample before formal testing, specifically identifying the maximum energy threshold for critical damage and the minimum energy threshold for any damage to occur. The energy value with relatively dense energy distribution is used to irradiate the sample, collect as rich data as possible, and provide a larger sample space for subsequent data processing. MATLAB R2022a is used to fit the sample’s LIDT using the least squares linear method, with energy density on the horizontal axis and damage probability on the vertical axis. The intersection of the line with the horizontal axis indicates the energy density at zero damage probability, representing the sample’s LIDT. The LIDT of a sample can be determined, but it usually has a wide range of uncertainties. When two samples have nearly identical LIDT values, their laser damage resistance is difficult to differentiate. It is essential to minimize LIDT uncertainty by analyzing its sources, conducting quantitative calculations, and enhancing measurement methods.

### 2.3. Methods for the LIDT Uncertainty

Insufficient knowledge of actual measurement errors can lead to inaccurate conclusions or inadequate safety factors when designing laser systems. We systematically analyze the test system’s error sources one by one. In the laser damage threshold test system, errors in determining the laser damage threshold mainly come from inaccuracies in laser parameters, energy meter accuracy, the damage identification process, laser energy density calculation, damage probability estimation, and data fitting. Compared with the errors of the latter three, the first two errors are very small and can be ignored. The damage identification error is a major error, which is mainly avoided by eliminating misjudgments. Therefore, the uncertainty of the LIDT is mainly composed of three parts: the laser energy density, the damage probability, and the data fitting. The following analysis discusses these three error sources.

#### 2.3.1. Energy Density Uncertainty u_crel_ (q)

The energy density q (J/cm^2^) of laser irradiation on the surface of the optical element is defined as the ratio of the laser energy Q (J) to the area of the laser spot πr^2^ (cm^2^), as shown in Formula (1). Here, “r” represents the radius of the spot deposited on the surface of the sample.

(1) $$q=\frac{Q}{\pi r^{2}}$$

In Formula (1), energy Q and radius r are the two variables, and there must be an error between the actual energy and the standard energy emitted by each laser. Similarly, there is an error between the actual spot radius and the calibration radius of the laser irradiation on the sample surface. Therefore, partial differentiation of Formula (1) gives Formula (2). Formula (2) shows that the energy density error, dq, is obtained from the partial differentiation of the energy density formula (Formula (1)).

(2) $$dq=\frac{1}{\pi r^{2}}dQ-\frac{2Q}{\pi r^{3}}dr=\frac{1}{\pi r^{2}}Qf-\frac{2Q}{\pi r^{3}}dr$$

Here, “f” represents the energy interval percentage of the energy level. To make the energy density error “dq” equal to 0, the following relationship must be satisfied:

(3) $$f=\frac{2}{r}dr$$

The experimentally calibrated laser has a spot radius of 400 μm with a ±10 μm error. If the energy density error (dq) is 0, then f is ±5% [31]. To calculate the damage probability within a specific energy interval, test points are selected at an energy level with a 5% positive or negative deviation from the standard energy, effectively minimizing uncertainty contributions.

#### 2.3.2. Combined Uncertainty of the Damage Probability u_crel_ (p)

The sample errors decrease indefinitely with the infinite increase in the number of experiments, according to statistics. However, the experimental data with finite energy levels (n) are usually used as samples in the actual test, which will inevitably affect the accuracy of the laser damage test results.

When calculating the damage probability in a certain energy level range, the standard energy density q is used to calculate the damage probability, which makes the actual damage probability uncertain (u_rel_(p_i_)), as shown in Formula (4) [34],

(4) $$u_{rel}(p_{i})=u(q_{i})/(\sqrt{k_{i}}\times {\bar{L}}_{i})$$

where u(q_i_) represents the uncertainty of the energy density. It is defined as the difference between the actual energy density and the standard energy density. k_i_ represents the number of measured points at the ith energy level, while $\bar{L}$_i_ denotes the average energy density at that level.

The energy emitted by the laser each time is not influenced by the previous emission. This means that the various energy densities are independent of one another, as is the uncertainty of damage probability at each energy level. Consequently, the total uncertainty of the damage probability u^2^_crel_(p) across all energy levels is determined by Formula (5), where n represents the total number of energy levels.

(5) $$u_{crel}^{2}(p)=\sum_{i=1}^{n}u_{rel}^{2}(p_{i})$$

#### 2.3.3. Data Fitting Uncertainty u_crel_ (L_th_)

According to the standard ISO 21254, the energy density of zero-probability damage is determined as the LIDT. Using the method of least squares, the fitted line intersects the coordinate axes through extrapolation, and the corresponding energy density at a probability of 0 damage is the LIDT of the sample. The linear regression equation is shown in Formula (6),

(6) $$\hat{L}=a\times p+b$$

where a is the regression coefficient of the linear regression equation, b is the energy density at the damage site with zero probability, and p is the standard energy density. The standard energy density value is considered the x coordinate, while the damage probability for each energy level is considered the y coordinate. The formula for calculating the uncertainty of linear fitting is presented in Formula (7) [35],

(7) $$u_{crel}(L_{th})=s(b)/b$$

where s(b) and s(L) represent the zero-probability damage standard uncertainty and the energy density standard uncertainty, respectively; Formulas (8) and (9) determine this.

(8) $$s(b)=s(L){\left\{n^{-1}+{\bar{p}}^{2}{\left[\sum_{i=1}^{n}{\left(p_{i}-\bar{p}\right)}^{2}\right]}^{-1}\right\}}^{1/2}$$

(9) $$s(L)={\left[{\left(n-2\right)}^{-1}\sum_{i=1}^{n}{\left({\bar{L}}_{i}-L_{i}\right)}^{2}\right]}^{1/2}$$

where L_i_ represents the standard energy density when the damage probability is p_i_, and $\bar{p}$ represents the average damage probability. The uncertainty of the linear fitting impacts the LIDT result.

#### 2.3.4. Relative Synthetic Uncertainty u_rel_ (F_th_)

In the laser-induced damage test system, the factors that affect the laser-induced damage threshold uncertainty are independent of each other, so the square of the LIDT standard uncertainty u_crel_ (F_th_) is equal to the sum of squares of each relative uncertainty, as shown in Formula (10) [35,36],

(10) $$u_{crel}(F_{th})={\left[u_{crel}^{2}(q)+u_{crel}^{2}(p)+u_{crel}^{2}(L_{th})\right]}^{1/2}$$

The extended uncertainty formula is shown in Formula (11),

(11) $$u_{rel}(F_{th})=cu_{crel}(F_{th})$$

In the formula, c represents the confidence coefficient, ranging from 2 to 3. For a confidence level of 0.95, c equals 2, and for a confidence level of 0.99, c equals 3.

## 3. Calculation Results and Analysis

Table 1 shows the original data and the calculated results of the damage probability. Based on prior analysis and methods, each uncertainty has been calculated, yielding the following results.

### 3.1. u_crel_ (L_th_) for the Sample

The experimental data were fitted using the least square method and MATLAB programming to obtain the linear regression equation y = 0.4683x − 7.1821, with coefficients a = 0.4683 and b = −7.1821. According to the definition of the LIDT, the LIDT = 15.34 J/cm^2^. The uncertainty calculation results for the sample are presented in Table 2, using the linear fitting formula.

### 3.2. u_rel_ (F_th_) for the Sample

The results showing the uncertainty of the laser-induced damage threshold for the sample are presented in Table 3. According to the previous analysis, when the energy error is 5%, the energy density uncertainty is 0.

### 3.3. The Result of the LIDT Fitting

**Figure 4:**
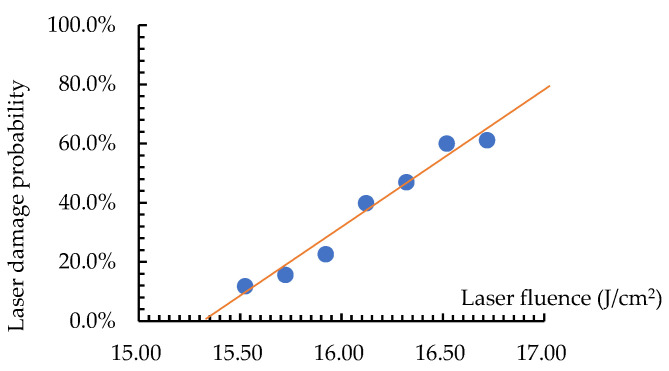
The result of the LIDT measurement for the sample.

The result of the LIDT fitting is shown in Figure 4. The structured surface optics with periodic cylindrical distribution has an LIDT of 15.34 ± 0.00052 J/cm^2^ at a 95% confidence level and 15.34 ± 0.00078 J/cm^2^ at a 99% confidence level. Multiple tests, each run over 100 times, guarantee high reliability with a minimal margin of error, boosting confidence in the LIDT measurement of this optical element. Periodic structured surfaces differ from traditional optical element surfaces due to their surface structures. During testing, ensure a narrow energy density interval and avoid abrupt divisions of equally spaced energy density. Repeat measurements as frequently as possible.

The anisotropic and complex surface structure necessitates an analysis of specific factors contributing to uncertainty. Each energy level is selected within a range of ±5%. We also examined other uncertainty factors primarily arising from the testing process. These procedures and data fitting enhance the accuracy of determining the LIDT of the surface. This study aims to provide a reliable data foundation for assessing the laser damage resistance of structural surfaces. This study demonstrates that we can be 95% and 99% confident in the true LIDT values within the specified range. It offers a reliable LIDT estimation for optical elements with a periodic cylindrical distribution, serving as an essential reference for performance evaluation and optimization in practical applications.

## 4. Conclusions

This paper investigates the LIDT uncertainty fitting method for optical elements with structural surfaces. We analyze the error sources in the test system and establish a calculation formula for LIDT uncertainty. The findings indicate that an appropriate energy error range and increased test repetitions can reduce uncertainty. We determined the LIDT of a two-dimensional grating with a periodic cylindrical distribution to be 15.34 ± 0.00052 J/cm^2^ (95% confidence level) and 15.34 ± 0.00078 J/cm^2^ (99% confidence level). These results provide a reliable basis for assessing the laser damage resistance of grating optical elements. This method is broadly applicable and can measure the LIDT in other periodic structures.

## Figures and Tables

**Table 1:** Results of the damage probability.

Item	*n*						
	1	2	3	4	5	6	7
f(%)	±0.05						
Standard energy(mJ)	78	79	80	81	82	83	84
Standard fluence Li (J/cm^2^)	15.53	15.73	15.92	16.12	16.32	16.52	16.72
${\bar{L}}_{i}$ (J/cm^2^)	15.81	15.58	15.81	16.24	16.38	16.54	16.54
u(q_i_) (J/cm^2^)	0.28	−0.15	−0.11	0.12	0.06	0.02	−0.18
k_i_	206	212	170	103	132	119	113
Damage ratio	12/206	18/212	19/170	41/103	62/132	67/119	69/113
P (%)	5.83	8.49	11.18	39.81	46.97	56.3	61.02
u_rel_ (p_i_)	0.00123	−0.00066	−0.00053	0.00073	0.00032	0.00011	−0.00102
u^2^_crel_ (p) (%)	0.000392						

**Table 2:** Results of the data fitting uncertainty.

Item	Uncertainty
s(L)	0.181
s(b)	0.123
u_crel_(L_th_)	−0.0171

**Table 3:** The results of each uncertainty calculation and the extended uncertainty.

Item	Uncertainty
u^2^_crel_ (q) (%)	0
u^2^_crel_ (p) (%)	0.000392
u^2^_crel_ (L_th_) (%)	0.000293
u_crel_ (F_th_) (%)	0.026
c = 2, u_rel_ (F_th_) (%)	0.052
c = 3, u_rel_ (F_th_) (%)	0.078

## Data Availability

The original contributions presented in this study are included in the article. Further inquiries can be directed to the corresponding author.
